# Current Strategies in Developing Antibacterial Surfaces for Joint Arthroplasty Implant Applications

**DOI:** 10.3390/ma18010173

**Published:** 2025-01-03

**Authors:** Giovana Collombaro Cardoso, Diego Rafael Nespeque Correa, Marco Fosca, Evgenii V. Pometun, Iulian V. Antoniac, Carlos Roberto Grandini, Julietta V. Rau

**Affiliations:** 1 Laboratório de Anelasticidade e Biomateriais, UNESP—Universidade Estadual Paulista, Bauru 17.033-360, SP, Brazil; diego.correa@unesp.br (D.R.N.C.); carlos.r.grandini@unesp.br (C.R.G.); 2 Istituto di Struttura della Materia, Consiglio Nazionale delle Ricerche (ISM-CNR), Via del Fosso del Cavaliere 100, 00133 Rome, Italy; marco.fosca@ism.cnr.it; 3 Department of Analytical, Physical and Colloid Chemistry, Institute of Pharmacy, I.M. Sechenov First Moscow State Medical University, Trubetskaya 8, Build. 2, Moscow 119048, Russia; pometun_e_v@staff.sechenov.ru; 4 Faculty of Material Science and Engineering, National University of Science and Technology Politehnica Bucharest, 313 Splaiul Independentei, District 6, RO-060042 Bucharest, Romania; antoniac.iulian@gmail.com; 5 Academy of Romanian Scientists, 54 Splaiul Independentei, RO-050094 Bucharest, Romania

**Keywords:** antibacterial coatings, antimicrobial coatings, surface treatments, antibacterial surfaces, implants, biomedical implants

## Abstract

Prosthetic joint infections (PJIs) remain a significant challenge, occurring in 1% to 2% of joint arthroplasties and potentially leading to a 20% to 30% mortality rate within 5 years. The primary pathogens responsible for PJIs include Staphylococcus aureus, coagulase-negative staphylococci, and Gram-negative bacteria, typically treated with intravenous antibiotic drugs. However, this conventional approach fails to effectively eradicate biofilms or the microbial burden in affected tissues. As a result, innovative strategies are being explored to enhance the efficacy of infection prevention through the development of antibacterial-coated implants. These coatings are required to demonstrate broad-spectrum antimicrobial activity, minimal local and systemic toxicity, favorable cost-effectiveness, and support for bone healing. In the present review, the analysis of various methodologies for developing antibacterial coatings was performed, emphasizing studies that conducted in vivo tests to advance potential clinical applications. A diversity of techniques employed for the development of coatings incorporating antimicrobial agents highlights promising avenues for reducing infection-related surgical failures.

## 1. Introduction

Improvements in routine perioperative antibiotic administration are effective in reducing the incidence of infection after orthopedic surgery. However, infections after knee and total hip arthroplasty are still one of the main causes of implanted biomaterial failure [1,2]. Prosthetic joint infection (PJI) occurs in 1% to 2% of all joint arthroplasties and can lead to death within 5 years in 20% to 30% of cases [3,4]. Infections also occur in patients who have undergone elective procedures (0.7 to 4.2%) or with exposed fractures (5 to 33%) [5,6].

**Figure 1:**
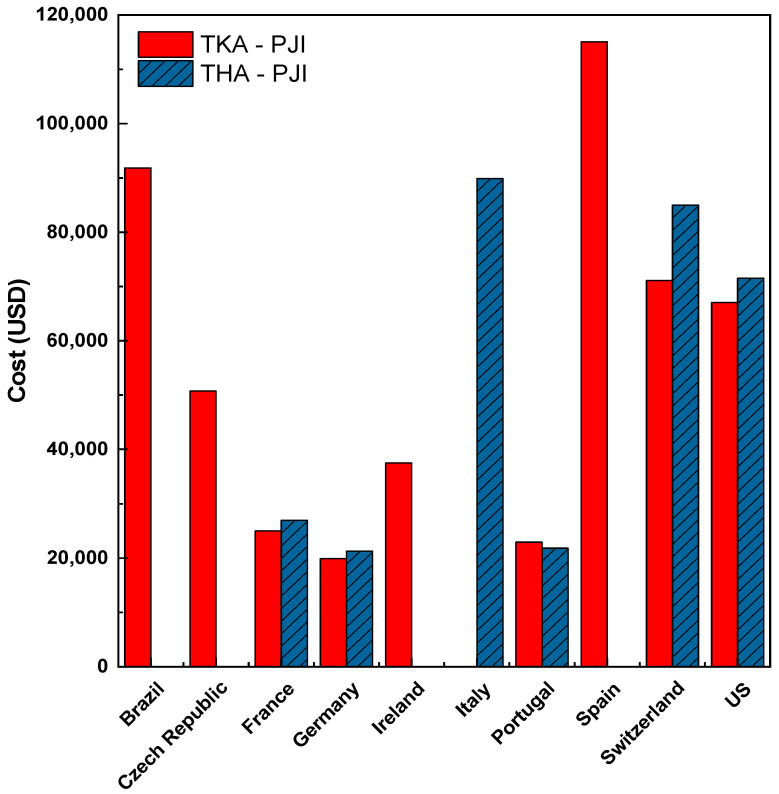
Costs associated with prosthetic joint infection treatment. TKA: Total Knee Arthroplasty; THA: Total Hip Arthroplasty [7,10,11].

Furthermore, the costs of treating PJI are high. Data indicate that the costs of treating PJI after hip and knee replacements can exceed USD 80,000 and USD 100,000, respectively [7,8,9]. The graph in Figure 1, created by the authors of this review, displays data from the literature on the costs associated with treating PJI after total hip arthroplasty (THA) and total knee arthroplasty (TKA) in several countries between 2008 and 2019 [7,10,11]. Likewise, some estimates show that the annual cost of treating PJI of the knee and hip will exceed USD 1.85 billion in the US [12,13].

Most prosthetic joint infections are caused by *Staphylococcus aureus*, coagulase-negative staphylococci, or Gram-negative strains and are treated with intravenous cefazolin [3,14,15,16]. However, this strategy is ineffective in eradicating biofilms from the materials or the *S. aureus* bioburden from the affected tissues [3]. PJI cases are common immediately after surgery. However, they can develop months or even years after the new material is implanted [17]. Therefore, strategies for obtaining materials with long-lasting antimicrobial activity must also be considered [9,18].

Thus, new strategies are being proposed to reduce the failure rate of surgeries caused by infections. These strategies involve promoting bactericidal properties to implant surfaces passively or actively [19]. Passive surface modifications aim to control bacterial adhesion by modifying surface topography, such as wettability and roughness [19,20]. On the other hand, active strategies aim to load the surface of implants with antibiotic or antiseptic agents [1]. Furthermore, the coatings would have to act against a broad spectrum of microorganisms, not present local or systemic toxicity, have excellent cost-benefit, and ensure bone healing [21].

Therefore, this review aims to analyze the literature on various strategies for developing bactericidal surfaces—an area of paramount importance for clinical applications, particularly in orthopedics. Our primary focus was on studies that report in vivo results, as these represent a critical step toward the clinical translation of newly developed materials. Given the limited number of in vivo studies, our reference list includes 102 selected sources, prioritizing those directly relevant to clinical outcomes.

The current literature predominantly addresses coatings and surface-modified implants. The following keywords were used to search the literature databases of published works: “antibacterial strategies”, “hip/joint”, and “arthroplasty/replacement”. After compiling the initial list of articles, they were reviewed, and only those presenting results from in vivo studies were selected. To organize our review, we divided the review into sections related to coatings and surface-treated implants with antibacterial properties. The first chapter is structured according to the types of bactericidal agents used in coatings, specifically antibiotics, and disinfectants. The second chapter provides an in-depth overview of both in vitro and in vivo tests performed on implants with functionalized surfaces. Finally, we explore future perspectives, focusing on potential developments and emerging trends in the field.

## 2. Antibacterial Strategies Applied on Coatings

Bactericidal coatings have emerged as a promising solution to prevent infections in hip and joint arthroplasty procedures, where bacterial contamination is a significant complication. In vivo studies are crucial to evaluating these coatings’ effectiveness in biological environments, offering better information about their ability to combat bacterial infections and promote implant osseointegration.

**Figure 2:**
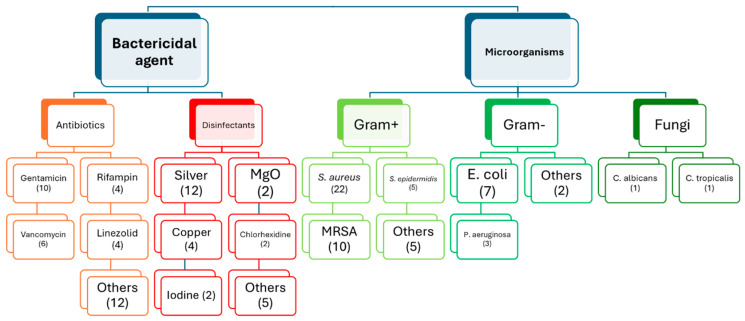
Organization of articles on bactericidal coatings for arthroplasties.

Therefore, this section focused on the literature review of implant coatings used in arthroplasties to determine their in vivo bactericidal efficacy. Figure 2 categorizes the collected papers based on the bactericidal agent and types of microorganisms tested.

Among antibiotics, ten articles studied the use of gentamicin [20,22,23,24,25,26,27,28,29,30], six used vancomycin [29,31,32,33,34,35], four worked with rifampicin [31,36,37,38], four with linezolid [31,36,37,39], and two with tobramycin [29,36]. The drugs daptomycin [31], berberine [40], ceftriaxone [36], cefazolin [36], cefepime [36], piperacillin [36], tazobactam [36], clindamycin [36], minocycline [38] and tigecycline [32] were used in only 1 study.

Another group of papers investigated the use of certain disinfectants in coatings as part of the bactericidal strategy. Twelve of them used silver [20,41,42,43,44,45,46,47,48,49,50,51], four studied the use of copper [52,53,54,55], two added magnesium oxide (MgO) [55,56], chlorhexidine [9,22] or iodine [57,58] to the coatings, and one paper used Cationic Steroidal Antimicrobial-13 (CSA13) [59], phage [39], Mouse Beta-Defensin-14 (MBD-14) [60], selenium [61], or XPerience (XP) [12].

The microorganisms used in the analyzed papers include Methicillin-Sensitive *Staphylococcus aureus* (MSSA) [12,20,23,25,26,28,30,31,32,34,35,36,38,40,42,43,54,55,56,57,60,62], Methicillin-Resistant *Staphylococcus aureus* (MRSA) [9,22,33,37,39,48,49,51,52,59], *Escherichia coli* [9,12,22,28,30,42,57], *Staphylococcus epidermidis* [9,12,22,40,51], *Pseudomonas aeruginosa* [12,22,60], Methicillin-Resistant *Staphylococcus epidermidis* (MRSE) [9], *Enterococcus faecalis* [12], *Cutibacterium acnes* [12], *Streptococcus pyogenes* [22], *Staphylococcus capitis* [53], *Enterobacter cloacae* [12], *Acinetobacter baumannii* [9], *Candida albicans* [12], and *Candida tropicalis* [12].

**Figure 3:**
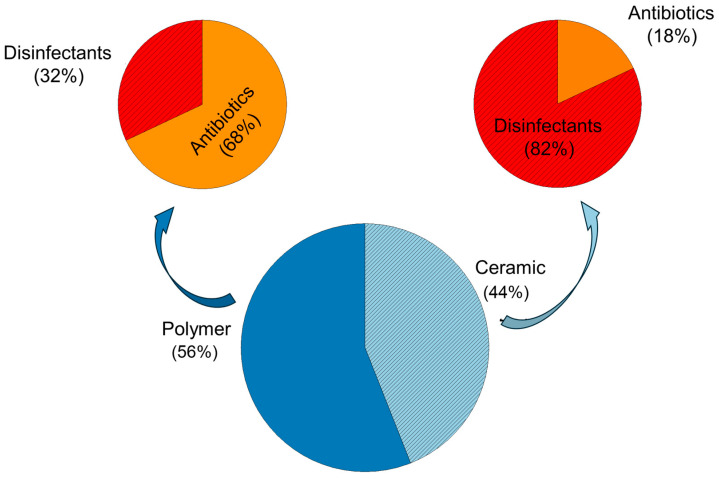
Quantification of articles relating to polymeric and ceramic coatings, and the most used bactericidal agents.

Finally, Figure 3 illustrates that, among the selected articles, 56% of the coatings were made of polymers and 44% of ceramics. Furthermore, among polymeric coatings, most bactericidal agents used were antibiotics (82%), while for ceramic coatings, more disinfectants were utilized (68%).

### 2.1. Use of Different Bactericidal Agents

#### 2.1.1. Antibiotics

The use of antibiotics before, during, and after surgery is a standard practice in orthopedic procedures [63]. Furthermore, research is being conducted on techniques for local antibiotic application to reduce the surgical infection rate. However, the presence of antibiotics around the implant is short-lived and may not be as effective against biofilm formation [32].

Using antibiotics loaded in coatings is advantageous as it reduces systemic toxicity [64]. However, using low doses of drugs for prolonged periods can be harmful, causing the development of drug-resistant strains [64]. Therefore, one strategy to prevent post-surgical infection is to improve the loading and release of drugs from the implant coatings.

**Figure 4:**
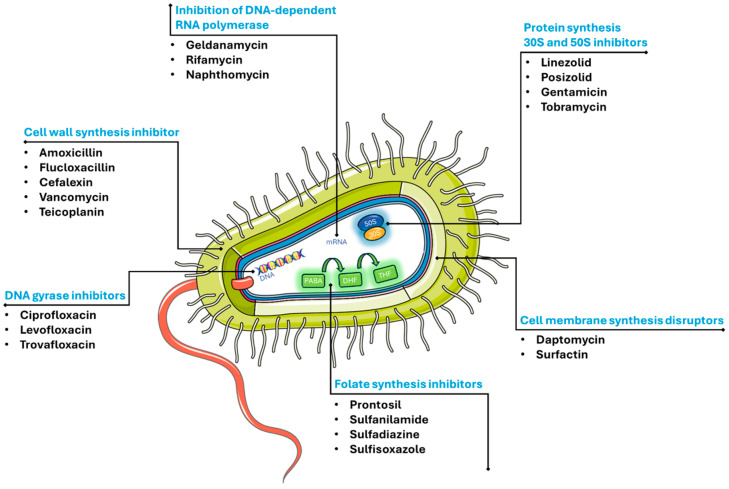
Mechanisms of action of different antibiotics. Parts of the figure were drawn using pictures from Server Medical Art. Servier Medical Art by Servier is licensed under a Creative Commons Attribution 3.0 Unported License.

Every antibiotic has a specific mechanism of action and targets different parts or processes of the bacterial cell, as illustrated in Figure 4 [65,66].

Gentamicin is an antibiotic known for its thermostability and efficacy in a broad bacterial spectrum, covering Gram-positive and Gram-negative bacteria [23,28,67,68]. Furthermore, it has good bone penetration [64] and helps prevent infections shortly after surgery [69], which makes it favorable for local antibiotic applications in orthopedic surgeries.

Another antibiotic used in biomedical implant coatings is vancomycin. It has bactericidal efficiency against many bacteria, including MRSE and MRSA [70]. Consequently, it is normally used to treat more serious infections caused by Gram-positive bacteria and prevent and treat osteomyelitis cases [71,72]. Furthermore, vancomycin is also widely used to treat osteoarthritis [35,73].

The third most used antibiotic among the selected papers was rifampicin. According to the literature, it is effective against *Staphylococcus* species [74,75] and is commonly used to treat early infections [76,77]. However, there is concern about the development of bacteria resistant to rifampicin when this antibiotic is used alone [31,37]. Therefore, one solution is to apply a combination of rifampicin and another type of antibiotic to the coatings [31,37].

Linezolid is a bacteriostatic agent that inhibits the formation of the initiation complex during bacteria’s protein synthesis phase [39]. Its use against infections after joint prosthesis placement is favored by its great efficacy against Gram-positive and drug-resistant strains, good tissue and bone penetration, and high bioavailability (it can be taken orally or intravenously) [39,78,79,80,81].

Regarding the use of gentamicin, Min et al. [25] studied polyetheretherketone (PEEK) implants coated with multilayers containing gentamicin and osteoinductive growth factor (BMP-2). The implants were placed in the tibias of adult male rats and tested against *S. aureus* Xen 29, which is 20 times more resistant to gentamicin than general strains. The antibiotic-treated group manifested little or no expression of *S. aureus* after 8 weeks post-revision. Furthermore, the growth factor led to over 80% bone coverage after 3 weeks of revision. Matsuno et al. [26] investigated coating Ti-6Al-4V, CoCrMo, and stainless steel with a hyaluronic acid gel containing gentamicin. The gel was chosen because it is bioabsorbable and biocompatible. In vivo studies showed a bactericidal effect up to 2 weeks after surgery. The authors presented results relating to 2 animal models. The mouse model results showed that the fractures were healed, and the gentamicin-containing material prevented osteomyelitis. In the rabbit model, the osseointegration of the implant was not impaired by the coating. However, the material did not appear to be effective against forming biofilms and other more serious cases of infection. Alt et al. [27,82] studied the effects of gentamicin on hydroxyapatite coatings produced on steel implants by an electrochemically assisted process. Rabbit model studies showed that the coatings effectively reduced S. aureus infection rates in 19 animals up to 4 weeks after surgery [82]. The authors also showed no significant difference in bone formation between the implant without and with gentamicin [27]. However, they observed a trend towards decreased new bone formation after 4 and 12 weeks of surgery with coatings containing gentamicin [27]. This may be because antibiotics, such as gentamicin, have been associated with impairments in the functioning of osteoblasts, influencing the osseointegration of implants [83,84,85,86].

Another group of authors studied the effects of vancomycin on its coatings. Stavrakis et al. [32] developed a biodegradable poly(ethylene glycol)-poly(propylene sulfide) (PEG-PPS) coating that can be used for antibiotic delivery. The authors tested vancomycin and tigecycline as antibiotics loaded in the produced coating and used titanium as the material to be coated. The in vivo results showed the great efficiency of the coatings in preventing the colonization of *S. aureus* on the implant’s surface and the surrounding bone and joint tissue after surgery. However, the results presented were better when using tigecycline. Furthermore, the bactericidal efficacy of this coating is limited to a short period after implantation. This is because the coating was designed to be biodegradable within 14 days. This way, it does not interfere with the osseointegration of the implant and does not provide an additional surface for bacteria to lodge. Giavaresi et al. [33] studied the bactericidal efficacy of a bioresorbable hydrogel coating loaded with vancomycin. In vitro results determined that over 80% of the vancomycin was released in the first 24 h. In vivo tests showed that the coating significantly reduced the MRSA bacterial load at the implant/bone interface after 7 days of surgery. Boot et al. [34] analyzed the bactericidal efficiency of a hydrogel coating loaded with vancomycin and two other non-antibiotic agents (bioactive glass (BAG) and N-acetyl-L-cysteine (NAC)) against *S. aureus*. This study placed hydrogel coatings on the surface of titanium implants. In vivo results showed that vancomycin-loaded coatings reduced the severity of infections and improved bone-to-implant contact. Compared to the hydrogel-implanted group, the BAG- and NAC-loaded coatings did not significantly reduce infection after surgery. However, applying local antibiotics was insufficient to eliminate the entire bacterial load. Fang et al. [35] produced a hybrid layer on the surface of an acetabular cup made of ultra-high molecular weight polyethylene (UHMWPE), using the traditional hot-pressing process and acid treatment to bond with a porous titanium layer loaded with vancomycin to evaluate the method’s efficiency against the bacterial activity of *S. aureus* for 7 days after subcutaneous implantation in rats. The authors demonstrated that the number of bacteria following the in vivo testing period was two orders of magnitude lower when the implant contained vancomycin than when it did not. Furthermore, a parallel set of tests conducted on Labrador dogs demonstrated biocompatibility and new bone-formation capability after 3 months. The authors highlighted that this biofunctionalized polymer can produce promising benefits when used in artificial joint prostheses at long-term implantation.

Among the studies analyzed, several investigated the combination of different medications. Ashbaugh et al. [31] developed a polymeric coating of poly(lactic-coglycolic acid) (PLGA) and poly(ε-caprolactone) (PCL) loaded with different combinations of antibiotics (rifampicin + vancomycin or rifampicin + linezolid or rifampicin + daptomycin) via electrospinning and applied to titanium wires. All combinations effectively prevented biofilm formation on the implant and inhibited bone changes caused by infections. Nevertheless, the combinations of rifampicin + linezolid and rifampicin + daptomycin had better efficacy due to a possible reduction in the bactericidal activity of vancomycin in biofilms with increased cell wall thickness. Miller et al. [37] produced a PLGA and PCL nanofiber coating loaded with a combination of rifampicin and linezolid via electrospinning. Nanofibers were used to coat titanium implants and used in rabbit surgeries. The authors measured the width of the mice’s knees 7 days after surgery to determine infection-induced inflammation. There were no changes in the animals treated with the implant loaded with antibiotics, while the group without antibiotics showed a 36% increase in the knee. They also found that the bioluminescence signals of MRSA were significantly greater when using implants without antibiotic loading.

Several advances have been made towards the development of new antibiotic-loaded coatings for orthopedic implants, mainly aimed at preventing post-surgical infections while, at the same time, promoting osseointegration of the implant. The reviewed studies highlight the potential of materials such as hydrogels, polymers, and composites to enhance the localized release of antibiotics. However, challenges persist in the field, including the limited duration of bactericidal efficacy, potential adverse effects on cellular activity, and the risk of developing antibiotic-resistant bacterial strains. Although combinations of antibiotics, such as rifampicin and linezolid, show promising results in combating biofilms and resistant bacteria, further studies are needed to optimize drug-loading techniques.

#### 2.1.2. Disinfectants

Another strategy for manufacturing bactericidal coatings is the release of bioactive ions, such as magnesium, silver, copper, zinc, and strontium. These ions help promote osseointegration, tissue regeneration, and vascularization [42,87,88,89].

**Figure 5:**
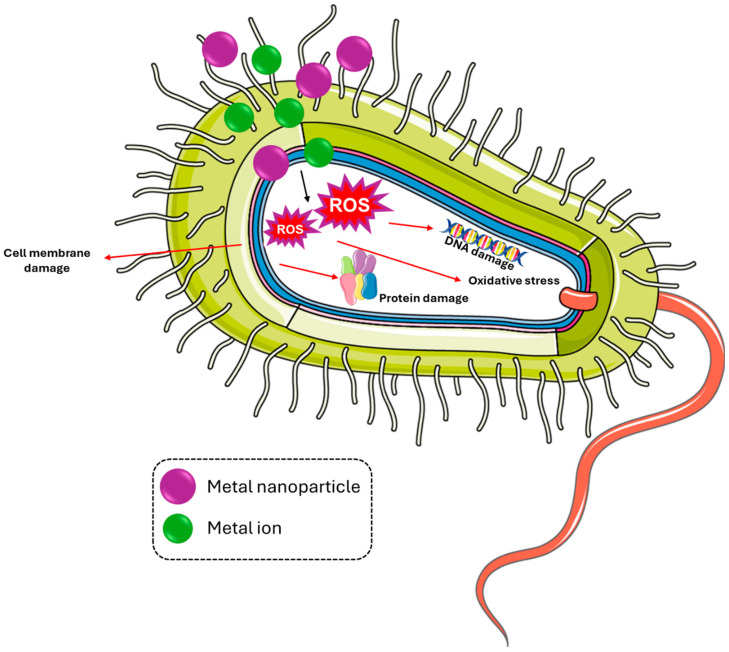
Mechanisms of action of bactericidal metal ions and metals nanoparticles. Parts of the figure were drawn using pictures from Server Medical Art. Servier Medical Art by Servier is licensed under a Creative Commons Attribution 3.0 Unported License.

In general, metal ions and nanoparticles penetrate and destroy the cell membrane, which can lead to the formation of reactive oxygen species (ROS), which damage proteins and DNA and induce oxidative stress [90,91], as illustrated in Figure 5.

Silver ions can neutralize the colonization of microorganisms on the surface of implants, which results in the non-formation of biofilm [92]. This occurs due to the interaction of ions with the DNA and metabolic enzymes of microorganisms, leading to difficulty in replication and cell death [93,94,95,96]. Silver has high toxicity to a wide spectrum of bacteria and a low risk of developing resistance [42,97,98]. Therefore, very small amounts of this agent are sufficient for the bactericidal effect to be manifested, which reduces the risk of toxic effects against human cells [92,97,99].

Copper is another metal widely used for its bactericidal effects against both Gram-positive and Gram-negative bacteria [100,101]. It has already been reported that copper has a “contact-killing” effect on bacteria, leading to cytoplasmic membrane damage and DNA degradation [52,100,102]. Furthermore, copper stimulates osteogenesis and angiogenesis, making it a good bone-formation agent [52,103].

Magnesium is an essential element for the human body, contributing to the formation of new bones [56,104]. However, magnesium is also important for bacterial metabolism, making its use difficult in implants requiring bactericidal properties [56]. One way forward is the use of magnesium oxides, which are alkaline and thus induce bacterial oxidative stress, killing microorganisms [105,106]. Furthermore, MgO increases osteogenesis and bone remodeling processes [55].

**Figure 6:**
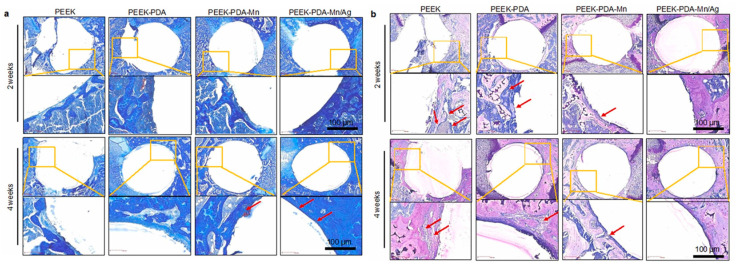
Masson (**a**) and Giemsa (**b**) staining of tissues around the implants after 2 and 4 weeks of implantation. In (**a**), the red arrows indicate the newly generated bones with low fibrous tissue, and in (**b**) indicate the chronic inflammatory cells in the interface of PEEK without Ag [42]. Reproduced with permission from Colloids and Surfaces B: Biointerfaces, 2023, 224, 113196, Elsevier.

Yang et al. [42] incorporated silver and manganese ions into the surface of a PEEK material. Two weeks after implantation of the material into *S. aureus*-infected mice, the interface of the PEEK material containing manganese and silver contained the greatest amount of new bone compared to materials containing only manganese or without any metallic components. Furthermore, histological analysis using Masson’s trichrome staining (Figure 6a) and Giemsa staining (Figure 6b) revealed that no infection occurred around the silver-containing implant, and fewer fibrous tissues appeared at the interface with the bone.

Zeng et al. [43] tested the bactericidal effect of a TiO_2_ nanotube coating containing Ag_2_O nanoparticles on animals infected by *S. aureus* after artificial joint replacement surgery. The results showed that the silver-containing implants effectively controlled the infection and presented excellent bactericidal properties. Eto et al. [46], Akiyama et al. [48], and Shimazakiet et al. [49] prepared silver-containing hydroxyapatite coatings on titanium via the thermal-spray method. The results showed low osteoconductivity of the uncoated implant surface after two weeks of implantation, unlike the coated surfaces [46]. Furthermore, the number of viable MRSA was significantly lower for silver-containing implants after 72 h of surgery [48,49]. Devlin-Mullin et al. [51] developed titanium scaffolds using selective laser melting (SLM) and covered them with a silver nanolayer via atomic layer deposition (ALD). Their in vivo results show that the implants induced osteogenesis and angiogenesis, showing no evidence of toxicity.

Regarding the use of copper, Huo et al. [52] developed a coating via electrophoretic deposition (EPD) composed of chitosan and a bioactive copper-doped glass. The authors also used titanium implants to be covered by the material. All groups of rats implanted with copper-containing materials showed less destruction of the femur after 3 weeks of surgery. Copper implants also reduced the severity of implant-related infections observed in rats implanted with pure titanium. Finally, the bacterial load in rat femurs was also reduced when copper-containing implants were used. Mauerer et al. [54] investigated the bactericidal properties of a Ti-6Al-4V spacer coated, via dip coating, with TiO_2_ doped with copper. After revision surgery, the insertion of copper-containing spacers demonstrated good results against MRSA infection.

Finally, Tan et al. [56] developed a coating on titanium via magnetron sputtering, containing Ca-O-Ti in the inner layer and MgO in the outer layer. After 6 weeks of implantation, the MgO-containing materials showed no obvious bone destruction and only a small amount of bacteria adhered to the coating. On the other hand, femurs implanted with uncoated titanium showed clear signs of infection. Finally, the MgO-coated implants were almost completely covered by new bone.

This review highlights the growing body of research on bactericidal coatings, which aim to play a critical role in improving implant success rates. In this scenario, silver, copper, and magnesium oxide emerge as three of the leading candidates due to their bactericidal properties, integration into implant materials, and considerable long-term stability. For example, silver’s low resistance to bacterial growth and broad-spectrum efficacy are advantageous features, but its cytotoxic potential requires more precise control over ion release after implantation. Similarly, copper’s role in combating infections and improving osteogenesis makes it a promising candidate. However, further studies are needed to establish an optimal dosage and its long-term effects. The literature reviewed in this section highlights the number of bactericidal agents used in implant coatings, including antibiotics such as gentamicin, vancomycin, and rifampicin and disinfectants like silver, copper, and magnesium oxide. The microorganisms tested in these studies span a wide spectrum, with a particular focus on common pathogens such as *Staphylococcus aureus*, *Escherichia coli*, and *Methicillin-Resistant Staphylococcus aureus*, underscoring the need for coatings that can effectively combat both Gram-positive and Gram-negative bacteria.

Additionally, the analysis of polymeric and ceramic coatings reveals distinct trends in bactericidal strategies. Polymeric coatings predominantly utilized antibiotics (82%), while ceramic coatings were more likely to incorporate disinfectants (68%), suggesting different approaches to achieving long-lasting antimicrobial effects based on the coatings’ material properties.

In conclusion of this section, while significant progress has been made in the development of antibacterial coatings for joint arthroplasty, further research, particularly in clinical trials and more advanced in vivo models, is essential to optimize these coatings for both infection prevention and enhanced implant integration. Future studies should continue to explore novel bactericidal agents and material combinations to improve the overall effectiveness of these coatings in clinical practice.

## 3. In Vitro and In Vivo Experimental Approaches for Surface-Treated Implants with Antibacterial Properties

### 3.1. In Vitro

New strategies for antimicrobial surface coating and treatment of biomaterials have been extensively reported in the literature. The studies mainly focused on using metallic materials for implants and conventional commercial techniques, such as those evolving mechanical, electrochemical, physical, or plasma methods, to produce bacteriostatic or bactericidal surfaces. Furthermore, in vitro antimicrobial activity and biofilm formation testing have been the first choices for the initial screening of the bacteria and surface interaction.

Yoda et al. [107] investigated the adhesion ability of *S. epidermidis* as distinct levels of surface roughness using in vitro tests. The study grouped distinct commercial metallic biomaterials, such as oxidized zirconium-niobium (Oxinium), cobalt-chromium-molybdenum alloy (Co-Cr-Mo), Ti-6Al-4V alloy, commercially pure titanium grade 2 (CP-Ti), and stainless steel (SUS316L). The surface roughness was altered by polishing with diamond slurry, and the samples were later divided into fine (Ra~10 nm) and coarse (Ra~30 nm) groups. The in vitro tests conducted after 48 h of incubation demonstrated that *S. epidermidis* tended to have better adhesion in the course group than the fine one. However, the Co-Cr-Mo sample’s hydrophobic behavior contributed to lesser bacteria adhesion when compared to the other biomaterials. The authors pointed out that this study shed some light on the minimal level of roughness needed to provide a reasonable impact on the bacteria adhesion in metallic implants. At the same time, Ma et al. [108] studied the potential usage of TiO_2_ nanotubes for drug delivery systems of antimicrobial peptides (AMPs). The nanotubes were grown on CP-Ti grade 2 using anodizing treatment at 30 V for 6 h in an ethylene glycol (C_2_H_6_O_2_) solution with 0.27 M of ammonium fluoride (NH_4_F). Then, the AMPs were incorporated into the nanotubes by immersion of the anodized titanium samples in a phosphate buffer solution enriched with the AMPs. The results indicated that the anatase TiO_2_ had better efficiency for the AMP loading, with drug release having a significant effect on reducing the activity and adhesion of *S. aureus* in the in vitro tests. Moreover, Guastaldi et al. [109] investigated the biological effects of the Ti-15Mo (wt.%) alloy surface after laser-beam irradiation. The surface was treated using a Yb:YAG laser beam, operating with a density power of 1.9 J·cm^−2^, pulse frequency in the range of 20 and 35 kHz, and exposure area of 14 mm^2^. The in vitro tests indicated an expressive effect on the adhesion of methicillin-resistant *Staphylococcus aureus* (*S. aureus*) when compared to the commercially pure titanium (CP-Ti) grade 2 treated under the same conditions. The authors stated that the laser-beam irradiation produced a homogeneous surface micro-roughness, which affected the materials’ physicochemical properties and antibacterial performance of the materials. Finally, Zaatreh et al. [16] investigated the antibacterial ability of Mg-based coatings on the biomedical Ti-6Al-4V alloy produced by magnetron sputtering. The results indicated that the level of biofilm and colony-forming units of *S. epidermidis* significantly decreased while the human osteoblastic cells had a pronounceable growth after 7 days of in vitro co-culture.

### 3.2. In Vivo

All the studies evolving in vitro testing have a consensus that further in vivo tests are needed for a better view of the benefits of these innovative surfaces for the next generation of biomedical implants. In this scenario, the scarce papers reporting in vivo antimicrobial testing are mainly focused on metals and polymers coated with a combination of bactericidal ions or antibiotics and their interaction with Gram-positive bacteria lineages.

For example, Ständert et al. [20] investigated the in vitro cytocompatibility, as well as the in vivo biocompatibility and antimicrobial ability of CP-Ti grade 5 samples submitted to a laser treatment and enrichment of Ag particles and gentamicin. The authors produced a micro-scaled porous surface using a Nd:YAG laser at 100 W, then used a sputtering chamber to embed Ag particles on the top of the pores and finally loaded the inner region of the pores with gentamicin. The in vitro results indicated the surface-treated samples possessed a unique combination of proper cell viability and differentiation of primary human osteoblastic cells, evaluated after 3 to 4 days of incubation, and enhanced osteointegration ability, drug-release ability, and antimicrobial effectivity against *S. aureus* after 3 days of culturing. However, the in vivo tests performed in Sprague–Dawley rats after 28 days of surgery indicated a clear reduction of histopathological bone destruction signs and the absence of bacteria colonization or infection of *S. aureus*. The authors point out that this surface treatment methodology can also be applied to other kinds of antibiotics, being a useful tool to fight against the implant-associated infections of orthopedical implants and prostheses.

Liu et al. [23] exploited the antibacterial effect and bone cell biocompatibility of nanotubular anodized titanium loaded with gentamicin using in vivo tests. The nanotube array was prepared by electrochemical anodization method performed in 0.09 M NH_4_F added to ethylene glycol with 10% water at 60 V for 30 min. The in vivo tests were conducted in New Zealand White rabbits during 6 weeks of implantation. The results indicated that the surface-treated samples diminished implant-related osteomyelitis and improved bone biocompatibility. Furthermore, the surface-treated samples depicted less bacterial growth of *S. aureus* than the bare material, with the potential to prevent local infections for joint replacement surgeries.

Considering now the biopolymers, there are also outstanding contributions regarding in vivo testing of antimicrobial surfaces. For example, Sang et al. [28] coated the surface of polyetheretherketone (PEEK) with silk protein-gentamicin and enriched the bulk with SrCO_3_ nanoparticles using traditional processing methods of chemical immersion. In vivo tests in Sprague–Dawley rats indicated strong osteogenic activity and antibacterial abilities against *S. aureus*, being further confirmed by in vitro tests. The authors noticed that this new innovative approach for surface modification of the PEEK can be useful for processing a novel generation of orthopedic implants. In the study of Sang et al. [40], the same strategy was used to coat PEEK with silk fibroin and load the bulk with osthole and berberine compounds. The in vivo tests performed in Sprague–Dawley rats for 10 weeks indicated that the samples possessed adequate osteogenesis, prevented endophytic infection, avoided bacterial adhesion of *S. aureus*, and exhibited a significant killing effect of suspended bacteria around it. The authors concluded that this biofunctionalized PEEK also has potential for use in orthopedy.

**Figure 7:**
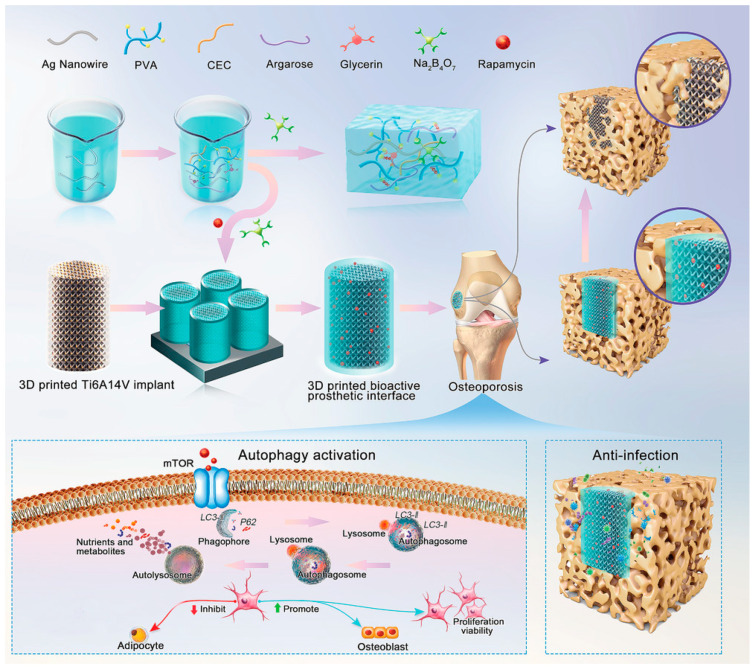
Illustrations of the bioactive and anti-infection 3D-printed titanium scaffold sample [110]. Reproduced with permission from Advanced Healthcare Materials, 2022, 11, 2102535, Wiley.

Li et al. [110] produced a novel hydrogel with a multi-hydrogen bond network structure enriched with polyvinyl alcohol, N-carboxyethyl chitosan, agarose, and silver nanowires (Figure 7). The material was produced by conventional sol–gel synthesis to provide innovative drug release and bone regeneration. After that, the hydrogel was embedded in a 3D-printed porous titanium alloy and evaluated in vivo in New Zealand White rabbits under the osteoporosis model after 3 months. The results were supported by in vitro tests, which confirmed the positive effect on cell proliferation, survival, osteogenic differentiation of bone mesenchymal cells, and the antibacterial effect against normal and methicillin-resistant *S. aureus*. The authors pointed out that this novel processing route can be an efficient strategy to produce novel prosthetic interfaces to avoid complications in patients with osteoporosis submitted to arthroplasty surgery.

Furthermore, some ongoing studies have also focused on the in vivo testing of hybrid materials produced by advanced processing methods. To cite, Ciliveri and Bandyopadhyay [55] pioneering employed metal additive manufacturing techniques (directed energy deposition and selective laser melting) to produce Ti-based samples enriched with MgO and Cu. Then, the authors evaluated the in vitro and in vivo biological aspects of the samples, focusing on their biocompatibility, osteogenesis, and antimicrobial aspects. The in vivo tests, conducted in Sprague–Dawley rats during 2 weeks of implantation, indicated that the MgO addition promptly stimulated bone mineralization and bone-implant strength, while the Cu presence promoted a pronounceable efficacy against *S. aureus* without evident cytotoxic effect. The authors highlighted that the designed samples can be useful for the manufacturing of advanced biomedical implants with superior osseointegration and antimicrobial capabilities.

It is possible to point out that the reduced number of in vivo antimicrobial testing studies has some drawbacks to be surpassed. For example, the studies are mainly focused on bactericidal strategies based on ion or drug release, despite bacteriostatic ones. Furthermore, they are restricted to small animal models (rats and rabbits) that still do not entirely reproduce the human body’s complex biological nature. Moreover, the studies only focused on Gram-positive bacteria, without evaluation of Gram-negative lineages or even other microorganisms (viruses and fungi). In this sense, it is important to know the current clinical needs in the experimental design of novel biomaterials. Alaee et al. [1] reported some important consensus related to orthopedic infections during the surgical procedure. The report details clinical issues related to the changing knife blade, surgical duration, operational room, antibiotic-coated implants, implant size and volume, C-arm contamination, robotics and computer-assisted surgeries, and patient-specific instrumentation. This kind of report is crucial to summarize the current understanding of the prevention of bacterial infection in patients submitted to implantation surgeries and should be considered in further research in the field.

To summarize this chapter, in vitro studies focused on various surface treatments, including mechanical, electrochemical, and plasma methods, aimed to create bactericidal surfaces. In vivo studies, however, remain limited but essential for validating these surface treatments. More in vivo studies, particularly in terms of using a wider range of bacteria, including also resistant hospital strains, are needed. Current studies predominantly use small animal models, which may not fully reproduce human conditions. Moving forward, these issues should be taken into account.

## 4. Conclusions and Future Prospectives

The growing concern about infections associated with biomedical implants has driven the development of new antimicrobial coating and treatment strategies for biomaterials.

Although in vitro studies have significantly advanced our understanding of the interaction between surfaces and microorganisms, the limited number of in vivo studies hinders the full validation of these innovations. In vivo testing is crucial for assessing the effectiveness of these coatings, offering valuable insights into their antibacterial properties and their ability to facilitate successful implant osseointegration.

After reviewing the selected articles, it can be concluded that although antibiotics are considered an effective approach to combat infections after implant placement, the risk of developing drug-resistant strains highlights the advantages of studying the use of bioactive ions.

The data obtained in this review suggests that, while the current focus is on conventional techniques and metallic materials, a more comprehensive approach should include the exploration of biopolymers and advanced hybrids. Recent research indicates that the use of TiO_2_ nanotubes, laser treatments, and coatings with biofunctionalized polymers, such as PEEK, offer promising antimicrobial properties and biocompatibility, although most investigations still focus on Gram-positive bacteria, such as *S. aureus*.

Furthermore, integrating clinical knowledge into biomaterials research is crucial for developing effective solutions that meet real-world needs in the surgical environment. Collecting data on operational factors and clinical complications associated with infections should guide the design of experiments that target both antimicrobial efficacy and long-term implant safety and functionality. Therefore, for new antimicrobial surfaces to be effectively implemented in clinical practice, a concerted effort to overcome the current limitations of biomaterials research is necessary, establishing a continuous dialogue between material science, clinical applications, and patient needs.

To advance this field, future research should focus on developing multidisciplinary approaches that integrate materials engineering, microbiology, and biomedicine. It is essential to investigate new combinations of antimicrobial agents and explore innovative surface modification methods that can inhibit bacterial adhesion without compromising biocompatibility. In addition, using more complex animal models representative of human physiology can provide more robust data on the efficacy and safety of new biomaterials. The application of emerging technologies, such as 3D printing and nanotechnology, can also open new frontiers in the design of personalized implants that meet the specific needs of patients. Finally, continued collaboration between industry, academia, and healthcare institutions will be essential to translate scientific discoveries into practical and innovative solutions for preventing infections in biomedical implants.

## Data Availability

No new data were created or analyzed in this study.
